# Tumor invasion in the central airway is a risk factor for early‐onset checkpoint inhibitor pneumonitis in patients with non‐small cell lung cancer

**DOI:** 10.1111/1759-7714.13703

**Published:** 2020-10-20

**Authors:** Mitsuhiro Moda, Haruhiro Saito, Terufumi Kato, Ryo Usui, Tetsuro Kondo, Yoshiro Nakahara, Shuji Murakami, Kouji Yamamoto, Kouzo Yamada

**Affiliations:** ^1^ Respiratory Medicine Tokyo Yamate Medical Center Tokyo Japan; ^2^ Department of Thoracic Oncology Kanagawa Cancer Center Yokohama Japan; ^3^ Department of Biostatistics Yokohama City University Yokohama Japan

**Keywords:** Central airway tumor invasion, drug‐induced pneumonitis, immune checkpoint inhibitors, non‐small cell lung cancer, risk factor

## Abstract

**Background:**

Anti‐programmed death‐1 (PD‐1) immunotherapy can cause immune‐related pneumonitis, also known as checkpoint inhibitor pneumonitis (CIP). CIP that develops early after the initiation of anti‐PD‐1 immunotherapy is important because it is more severe than CIP that develops later. However, only a few studies have examined the risk factors for early‐onset CIP. Previous studies have reported several risk factors for CIP, including imaging findings of airway obstruction adjacent to lung tumors. However, the utility of this factor is debatable. Therefore, we investigated potential risk factors for early‐onset CIP, including tumor invasion in the central airway (TICA), in patients with non‐small cell lung cancer (NSCLC) receiving anti‐PD‐1 therapy.

**Methods:**

We retrospectively analyzed the medical records and chest computed tomography scans of patients with NSCLC treated with anti‐PD‐1 antibodies at the Kanagawa Cancer Center in Japan between 1 January 2016, and 30 June 2018. The clinical characteristics and imaging findings, including TICA, were compared between patients with and without early‐onset CIP.

**Results:**

Data from 181 eligible patients (114 receiving nivolumab and 67 receiving pembrolizumab) were analyzed. Early‐onset CIP occurred in 13 of 79 patients (16.5%) with TICA and 2 of 102 patients (2.0%) without TICA. In multivariate analysis, the odds ratio of early‐onset CIP for patients with TICA was 8.2 (95% confidence interval [CI]: 1.98–34.0, *P* = 0.0037).

**Conclusions:**

TICA was strongly associated with early‐onset CIP in patients with NSCLC. Clinicians should carefully observe patients with TICA, especially within three months of anti‐PD‐1 antibody administration because of high CIP risk.

**Key points:**

**Significant study findings**

- Tumor invasion in the central airway (TICA) was a predictor of early‐onset checkpoint inhibitor pneumonitis (CIP)
- TICA had good interobserver variability, indicating its utility in clinical practice
- Patients with TICA might have a higher immune status than patients without

**What this study adds**

- This is the first study focusing on risk factors for CIP limited to early‐onset CIP.

## Graphical abstract

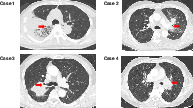

## Introduction

Anti‐programmed death‐1 (PD‐1) antibodies have demonstrated promising results against several cancers, including non‐small cell lung cancer (NSCLC),^1^, ^2^, ^3^, ^4^ resulting in a paradigm shift in patient treatment. However, anti‐PD‐1 immunotherapy can be associated with various immune‐related adverse events (irAEs).^5^ One such irAE, drug‐induced pneumonitis, also called checkpoint inhibitor pneumonitis (CIP), is important because of its incidence and severity.^6^, ^7^ Recent studies showed a higher incidence of CIP in the real‐world setting (7.2–14.6%) than those previously reported in clinical trials (3.6–4.1%).^8^, ^9^, ^10^, ^11^ Therefore, understanding the risk factors for CIP in the real world is vital.

Various studies have examined the risk factors for CIP.^7^, ^8^, ^9^, ^10^, ^11^, ^12^, ^13^ However, several risk factors remain controversial. Among them, the imaging findings of airway obstruction adjacent to lung tumors (IAOT), reported by Nakahama *et al*,^13^ is of interest. Nakahama *et al*. reported that IAOT was associated with an increased risk of CIP (odds ratio [OR], 6.59), a higher risk than a history of radiation pneumonitis, a well‐known risk factor for CIP, with an OR of 3.96. However, despite these interesting results, this association has not been validated, probably because of interobserver variability for the diagnosis of IAOT. We modified IAOT, which has no exact definition, to create a new imaging finding, which we named tumor invasion in the central airway (TICA) and defined it precisely to be more useful in the clinical setting.

Moreover, CIP that occurs early after initiation of anti‐PD‐1 immunotherapy is reported to have a higher severity than late‐onset CIP.^8^ However, there is no study focusing on the risk factors for early‐onset CIP, which is defined as CIP occurring within three months of the initiation of anti‐PD‐1 immunotherapy.

Therefore, we investigated potential risk factors for early‐onset CIP, including TICA, in patients with NSCLC receiving anti‐PD‐1 therapy.

## Methods

### Patient characteristics

We retrospectively analyzed the electronic medical records of patients with advanced or recurrent NSCLC after surgical resection or radiotherapy, who were prescribed anti‐PD‐1 antibodies (nivolumab or pembrolizumab) as routine practice outside of clinical trials at the Kanagawa Cancer Center between January 2016 and June 2018. Patients who did not undergo chest computed tomography (CT) in the two months preceding anti‐PD‐1 therapy were excluded.

All patients were administered at least one dose of nivolumab monotherapy (3 mg/kg or 240 mg/individual every two weeks) or pembrolizumab monotherapy (200 mg/individual every three weeks). The observation period was between 1 January 2016 and 31 December 2018. We obtained baseline patient characteristics, including age, gender, smoking history, histology, prior therapy (including surgery and radiotherapy), white blood cell count, lactate dehydrogenase, and C‐reactive protein (CRP).

In this study, we divided CIP into early‐onset CIP (defined as CIP that occurs within three months of initiation of anti‐PD‐1 immunotherapy) and late‐onset CIP (defined as CIP that occurs after >three months of initiation of anti‐PD‐1 immunotherapy) because the radiological finding of TICA (see next section) will be changed by the antitumoral effect; hence, TICA should be evaluated as a risk factor for CIP only early after immunotherapy initiation.

We equally collected data on the incidence of early‐onset CIP (defined as CIP that occurs within three months of the initiation of anti‐PD‐1 immunotherapy), as well as the grade of CIP and clinical course after CIP. The severity of CIP was graded using the Common Terminology Criteria for Adverse Events, Version 4.0.^14^ Clinical characteristics and imaging findings were compared between patients with and without early‐onset CIP to identify potential risk factors for early‐onset CIP. Moreover, CIP grade and clinical course were evaluated according to the presence or absence of TICA on CT. Additionally, to investigate the impact of TICA on the clinical course, progression‐free survival (PFS) and overall survival (OS) were analyzed according to the presence or absence of TICA. PFS was defined as the time between the day the baseline CT scan was performed and disease progression or death from any cause, and OS was defined as the time between the day the baseline CT scan was performed and death from any cause.

To compare the impacts of early‐ and late‐onset CIP (defined as CIP that occurs more after than three months of the initiation of anti‐PD‐1 immunotherapy) on the clinical course, we also investigated CIP grade and the clinical course of late‐onset CIP.

### Radiographic analysis

**Figure 1:**
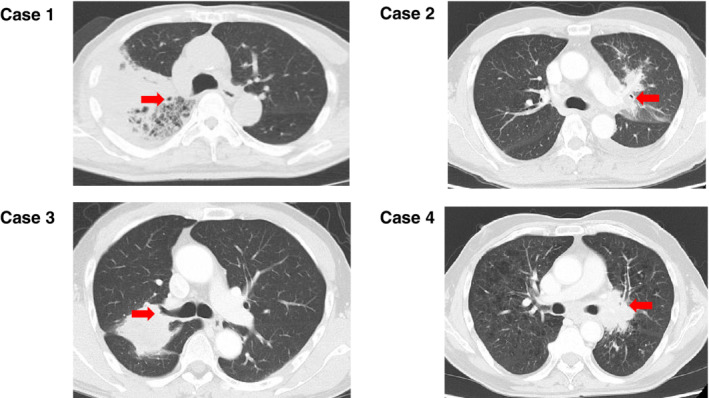
Representative imaging of tumor invasion in the central airway.

All patients in the present study were examined by a multidetector row CT scanner with a slice thickness of 5 mm and collimation of 5 mm within two months prior to anti‐PD‐1 therapy initiation. We defined TICA as the development of a tumor from the adjacent to the proximal portion of the main bronchus to the periphery of the segmental bronchus, resulting in a narrowing of the bronchial lumen to less than one‐quarter of the original volume or occurrence of an irregular bronchial wall (Fig 1). Analysis for the determination of the presence of pulmonary fibrosis, emphysema, mediastinal lymphadenopathy, pleural effusion, intrathoracic or extrathoracic metastasis, and TICA was performed by three pulmonologists blinded to the clinical data, with discrepancies resolved by consensus. In addition, we analyzed interobserver variability in the identification of TICA to evaluate reproducibility. The diagnosis of CIP was established by newly detected opacity in the lung parenchyma after other causes, such as pulmonary infections, pulmonary edema, and progressive cancer, were excluded by careful examination.^15^

### Statistical analysis

The demographic and clinical characteristics of patients who developed CIP were compared with those of patients who did not develop CIP using Student's *t*‐test for continuous variables and Fisher's exact test for categorical variables. Multivariate logistic regression analyses were conducted to evaluate the risk factors for early‐onset CIP among clinical and radiologic variables that were significantly different between patients with and without CIP. We used the shrinkage method to resolve overfitting because the number of candidate factors was too large for the number of events. A *P*‐value <0.05 was considered statistically significant. The Kaplan–Meier method and log‐lank test were used to display and compare, respectively, the PFS and OS in patients with or without TICA. Furthermore, for each observer, we calculated the Cohen κ value for interobserver agreement of the presence of TICA. The strength of interobserver agreement indicated with κ value was classified as follows: poor, κ was less than 0.0; slight, κ ranged from 0.0 to 0.20; fair, κ ranged from 0.21 to 0.40; moderate, κ ranged from 0.41 to 0.60; substantial, κ ranged from 0.61 to 0.80; and almost perfect, κ ranged from 0.81 to 1.00.^16^ We used the R software version 3.6.2 (R Foundation, Vienna, Austria) for the statistical analyses.

### Study ethics

The study was approved by the Institutional Review Board of the Kanagawa Cancer Center and complied with the principles of the Declaration of Helsinki. The need for informed consent was waived because of the retrospective nature of the study.

## Results

### Baseline characteristics of patients

**Figure 2:**
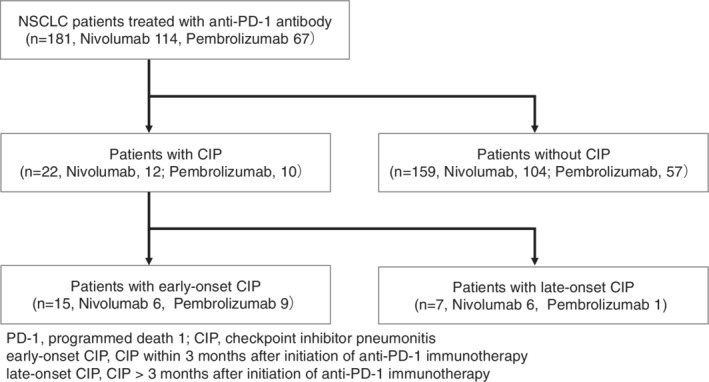
Patient flow chart of study.

There were 181 patients with advanced or recurrent NSCLC who were eligible, 22 (12%) of whom developed CIP during a median follow‐up period of 12.3 months. Among these 22 patients, 15 (8.3%) developed early‐onset CIP (Fig 2). The baseline characteristics of all patients and differences in characteristics between patients with and without early‐onset CIP are summarized in Table 1. The median age was 69 years (range, 38–85 years), 44 patients were women (24%), and 145 (80%) patients had a smoking history. A total of 105 patients (58%) had adenocarcinoma, 45 patients (25%) had squamous cell carcinoma, and 31 (17%) patients had a histology classified as “other.” In total, 114 (63%) and 67 (37%) patients received nivolumab and pembrolizumab, respectively. A total of 39 patients (22%) had a history of thoracic radiotherapy, and 79 patients (43%) had TICA on chest CT before with the initiation of anti‐PD‐1 antibody therapy. There were significant differences between patients who did and did not develop CIP with respect to more than two lines of chemotherapy (*P* = 0.013), CRP levels ≥1 mg/dL (*P* = 0.026), and TICA (*P* = 0.003).

**Table 1:** Baseline characteristics

	All patients *n* = 181	Patients with early‐onset CIP *n* = 15	Patients without early‐onset CIP *n* = 166	*P*‐value
Age	69 (38–85)	69.2 (59–81)	66.9 (38–85)	0.344
Male sex, *n* (%)	137 (75.7)	14 (93.3)	123 (74.1)	0.122
Current/ex‐smoker, *n* (%)	145 (80.1)	15 (100)	130(78.3)	0.078
Prior thoracic radiotherapy, *n* (%)	39 (21.5)	2 (13.3)	37 (22.3)	0.530
Prior lung resection, *n* (%)	53 (29.3)	1 (6.7)	52 (31.3)	0.071
Extra thoracic metastasis, *n* (%)	108 (59.7)	9 (60)	99 (59.6)	1.000
ICI agent: pembrolizumab, *n* (%)	67 (37.0)	9 (60)	58 (34.9)	0.090
Line of chemotherapy: ≧2, *n* (%)	136 (75.1)	7 (46.7)	129 (77.7)	0.013
Emphysema, *n* (%)	79 (43.6)	9 (60)	70 (42.2)	0.277
Lung fibrosis, *n* (%)	9 (5.)	1 (6.7)	8 (4.8)	0.549
Mediastinal lymphadenopathy, *n* (%)	91 (50.3)	10 (66.7)	81 (48.8)	0.281
Pulmonary metastasis: ≧4, *n* (%)	40 (22.1)	4 (26.7)	36 (21.7)	0.745
Pleural effusion, *n* (%)	58 (32.0)	3 (20.0)	55 (33.1)	0.393
WBC: ≧9000/μL, *n* (%)	36 (19.9)	5 (33.3)	31 (18.7)	0.183
Lymphocyte: ≧1500/μL, *n* (%)	61 (33.7)	7 (46.7)	54(32.5)	0.269
CRP: ≧1 mg/dL, *n* (%)	78 (43.1)	11 (73.3)	67 (40.4)	0.026
LDH: ≧240 IU/L, *n* (%)	62 (34.3)	5 (33.3)	57 (34.3)	1.000
Histology, *n* (%)				
Squamous	45 (24.9)	5 (33.3)	40 (24.1)	0.532
Adenocarcinoma	105 (58.0)	8 (53.3)	97 (58.4)	0.787
Others	31 (17.1)	2 (13.3)	29 (17.5)	1.000
Tumor invasion in the central airway, *n* (%)	79 (43.6)	13 (86.7)	66 (39.8)	0.003

Date are shown as median (range) or number (%).

CRP, C‐reactive protein; LDH, lactate dehydrogenase.

### Risk factors for early‐onset CIP

Based on previous reports^7^, ^8^, ^9^, ^10^, ^11^, ^12^, ^13^ and the analysis of baseline characteristics in the present study, five candidate factors (histology, a history of thoracic radiotherapy, TICA, number of lines of chemotherapy, and CRP levels) were included in the multivariate logistic regression analysis (Table 2). Considering the small number of patients with CIP, the number of candidate factors was relatively large; however, we used the shrinkage method to avoid overfitting. We found that only TICA (OR, 8.20; 95% confidence interval [CI]: 1.98–34.0, *P* = 0.0037) was associated with a significantly higher incidence of CIP.

**Table 2:** Multivariate odds ratios (ORs) for early‐onset checkpoint inhibitor pneumonitis

Risk factors	Comparison	OR(95.0% CI)	*P*‐value
Histology	Squamous vs. adeno	1.155 (0.323‐4.134)	0.825
Line of chemotherapy	First line vs. second line	2.914 (0.901‐9.425)	0.074
Prior thoracic radiotherapy	Yes vs. No	0.664 (0.137‐3.226)	0.612
CRP	1 mg/dL vs. <1 mg/dL	2.921 (0.842‐10.052)	0.090
Tumor invasion in the central airway	Yes vs. No	8.203 (1.980‐34.001)	0.004

CI, confidence interval; CRP, C‐reactive protein.

### CIP grade and clinical course

**Figure 3:**
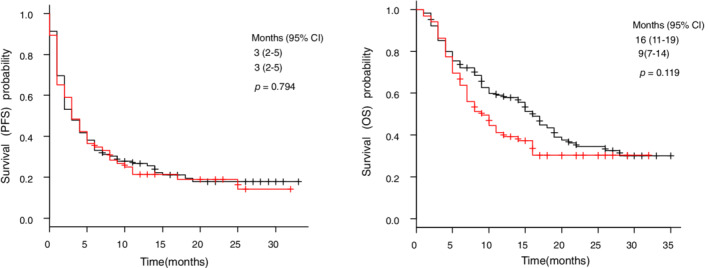
Kaplan–Meier curves for (**a**) PFS and (**b**) OS according to TICA. CI, confidence interval; OS, overall survival; PFS, progression‐free survival; TICA, tumor invasion in the central airway. (**a**) (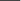) No‐TICA; (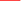) TICA. (**b**) (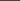) No‐TICA; (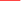) TICA

Detailed clinical characteristics of patients with early‐onset CIP are shown in Table 3. Early‐onset CIP occurred in 13 (16.5%) of 79 patients with TICA and two (2.0%) of 102 patients without TICA. Both patients (100%) without TICA developed grade 2 or lower CIP, whereas six patients (46%) with TICA developed grade 2 or higher CIP; two of these patients died due to CIP, despite treatment (Table 4). Moreover, both patients (100%) without TICA were able to undergo the subsequent round of chemotherapy, whereas seven patients (53%) with TICA were able to undergo the subsequent round of chemotherapy. However, in all patient groups, there were no significant differences in the PFS and OS between patients with and without TICA (Fig 3).

**Table 3:** Clinical characteristics of patients with early‐onset CIP

Baseline characteristics								CIP characteristics and clinical course		
Age	Sex	TICA	Histological type	Radiation	ICI	Therapy line	CRP value (mg/dL)	Time to onset (days)	CIP Grade	Next chemotherapy
62	M	Yes	Adeno	No	Pembro	1	3.51	21	2	Yes
74	M	Yes	Adeno	No	Pembro	1	8.55	64	2	Yes
70	M	Yes	Adeno	No	Pembro	1	7.52	2	5	No
73	M	Yes	Sq	No	Pembro	1	6.81	20	3	No
63	M	Yes	Others	No	Pembro	2	2.58	26	3	Yes
68	M	Yes	Adeno	No	Pembro	1	9.72	2	3	Yes
81	M	Yes	Adeno	No	Pembro	1	0.83	42	2	Yes
74	M	Yes	Others	No	Pembro	1	11.85	82	2	No
79	M	No	Adeno	No	Pembro	1	0.35	5	2	Yes
66	M	Yes	Adeno	No	Nivo	2	3.83	13	3	No
75	M	No	Adeno	No	Nivo	2	4.27	27	2	Yes
56	M	Yes	Sq	No	Nivo	2	4.36	5	5	No
62	M	Yes	Sq	Yes	Nivo	2	18.84	54	2	No
59	M	Yes	Sq	Yes	Nivo	2	0.45	82	2	No
77	F	Yes	Adeno	No	Nivo	2	0.06	28	2	No

Adeno, adenocarcinoma; CIP, checkpoint inhibitor pneumonitis; CRP; C‐reactive protein; Early‐onset CIP, CIP that occurs within three months after initiation of anti‐PD‐1 immunotherapy; F, female; ICI, immune checkpoint inhibitor; M, male; Nivo, nivolumab; Pembro, pembrolizumab; Sq, squamous cell carcinoma; TICA, tumor invasion in the central airway.

**Table 4:** Grade of checkpoint inhibitor pneumonitis (CIP)

		CIP grade				
		1	2	3	4	5
Early‐onset CIP *n* = 15	TICA+ *n* = 13	0	7	4	0	2
	TICA− *n* = 2	0	2	0	0	0
Late‐onset CIP *n* = 7		1	3	3	0	0

Early‐onset CIP, CIP within three months after initiation of anti‐PD‐1 immunotherapy; Late‐onset CIP, CIP >three months after initiation of anti‐PD‐1 immunotherapy; TICA, tumor invasion in the central airway.

Late‐onset CIP occurred in seven patients; however, all seven responded to corticosteroids and none died of CIP.

### Interobserver variability in the identification of TICA

The interobserver agreement among the three pulmonologists (MM, HS, and TK) for the identification of TICA on CT was substantial (κ value = 0.74).

### Discussion

In this study, we investigated risk factors for early‐onset CIP, a type of pneumonitis that occurs within three months of anti‐PD‐1 immunotherapy initiation. To the best of our knowledge, this is the first study focusing on risk factors for CIP limited to early‐onset CIP. Similar to the results of a previous study,^8^ in our study, early‐onset CIP caused death in two patients, whereas no patients died of late‐onset CIP, which implies that early‐onset CIP may be more severe than late‐onset CIP. Therefore, early‐onset CIP has a greater impact on a patient's clinical course and should be managed with greater care. Therefore, we thought it important to evaluate factors predictive of early‐onset CIP specifically.

TICA was associated with a significantly increased risk of early‐onset CIP. In a previous study by Nakahama *et al*., IAOTs were a potential risk factor for CIP; however, they lacked an accurate definition,^8^ which led to a difficulty in using this finding in clinical practice. Therefore, we modified IAOT to TICA, which is accurately defined, and validated the interobserver variability in the identification of TICA. The strength of the interobserver agreement was good, which indicates that this finding can be generalized and useful in clinical practice. Although TICA might be discussed in terms of bronchoscopy, we did not adopt endoscopic findings in TICA to prioritize its use in clinical practice.

All patients without TICA who developed early‐onset CIP could undergo the subsequent round of chemotherapy, whereas only 53% of patients with TICA who developed early‐onset CIP could undergo the subsequent round of chemotherapy. Although only two patients without TICA developed early‐onset CIP; this result may indicate that early‐onset CIP with TICA leads to a poorer prognosis compared to early‐onset CIP without TICA. Interestingly, a previous study^8^ also demonstrated that CIP grade was more severe in patients with IAOT than in those without IAOT. However, there were no significant differences in the PFS and OS between patients with and without TICA, thus indicating that we do not need to refrain from using anti‐PD‐1 immunotherapy in patients with TICA; however, careful observation for CIP occurrence is required in these patients.

There are several potential reasons why TICA may be a risk factor for early‐onset CIP. As mentioned in a previous study,^8^ since it is common for patients with airway obstruction to develop recurrent obstructive pneumonitis, we postulate that TICA may be a source of inflammation, which contributes to CIP development. Therefore, we defined TICA to include not only complete obstruction of the airway but also narrowing of the airway that could be a risk for obstructive pneumonitis. This differs from IAOT, which may only include complete obstruction of the airway. One major hypothesis of CIP onset is that cytotoxic T lymphocytes reactivated by anti‐PD‐1 antibody attack the normal lung. These T cells can share antigens with the tumor or cause a hyperimmune state in the tumor, and these factors influence the tissue around the normal lung.^17^, ^18^ However, some studies have demonstrated that retreatment with anti‐PD‐1 antibody after CIP is successful,^9^, ^19^ implying that there are other potential mechanisms of CIP development. The PD‐1 pathway has roles other than antitumor immunity, including negative feedback mechanisms to inhibit hyperinflammation.^19^, ^20^, ^21^, ^22^, ^23^ Several studies have reported an association between inflammation, including infection, and CIP. Baba *et al*. reported that nivolumab aggravates existing nontuberculous mycobacteriosis or *P. aeruginosa* infection and may cause inflammation from a subclinical pneumocystis pneumonia infection in some patients.^24^ Cui *et al*. reported that prior lung disease, including pneumonitis, is a risk factor for CIP.^12^ Kanai *et al*. reported that patients with rheumatoid arthritis, who are known to have a more inflammatory state in the lung and are more susceptible to bacterial pneumonia than healthy subjects, appeared to develop CIP more frequently, although the difference was not significant.^10^ Finally, Yamaguchi *et al*. reported that all patients with honeycombing who develop CIP show new ground glass opacities or consolidations superimposed on the honeycombing area that are known to comprise inflammatory cells and latent infection.^11^ Furthermore, to elucidate the association between TICA and inflammation, we compared CRP levels, which indicate systemic inflammation in the body,^25^ between patients with and without TICA. The proportion of patients with CRP ≥1 mg/dL was significantly higher among patients with TICA than among those without TICA (*P* = 0.003). This result implied that patients with TICA may have a pro‐inflammatory state. However, additional studies investigating the association between TICA and local or systemic inflammation are needed to elucidate the mechanism.

Interestingly, pre‐existing pulmonary fibrosis^10^, ^11^ and prior thoracic radiotherapy,^12^, ^13^ which are well known‐risk factors for CIP, were not significant risk factors in this study. There are two potential reasons for this. First, the number of patients with pulmonary fibrosis was very small, and there was insufficient power to identify significance. Second, prior thoracic radiotherapy has been reported to be a significant risk factor for CIP, but some reports do not show significance.^9^ This may be because of selection bias due to the retrospective nature of the studies.

In the present study, we divided CIP into early‐onset CIP and late‐onset CIP because the radiological TICA findings will be changed by immunotherapy; therefore, TICA should be evaluated only early after initiation of this therapy. Although there is no study validating the utility of classifying CIP according to the time of onset to initiation of immunotherapy, we think this classification is very important for clinical practice because of potential differences in the clinical course and steroid‐responsiveness of CIP. One study reported that most cases with steroid‐resistant pneumonitis occurred within three months of initiation of immunotherapy (71.4%), and most cases with CIP grade 5 occurred three months thereafter (75%).^8^ Another study also demonstrated that most cases with steroid‐resistant pneumonitis also occurred after three months (77%).^26^ In the present study, all cases with steroid‐resistant or grade 5 pneumonitis occurred around this period, while all cases with CIP that occurred after >3 months of initiation of anti‐PD‐1 immunotherapy responded to corticosteroids and none died of CIP. However, further studies are needed to determine the utility of this classification, appropriate time from initiation of immunotherapy to distinguish between “early‐onset” and “late‐onset,” and even potential differences in the pathogenesis of CIP.

We acknowledge several limitations of this study. First, the major limitation is the small sample size. In particular, the number of patients without TICA who developed early‐onset CIP was very small. Second, this study was retrospective at a single center, and this might cause potential bias. We tried to address this by using multivariate analysis to adjust for confounding factors. Finally, this study only includes Japanese patients. As drug‐induced pneumonitis appears to be more frequent in Japanese patients,^6^, ^27^ the results of this study may not be fully applicable to different ethnic groups. Despite these limitations, this study offers clinicians a relationship between CIP and TICA, which may be easier to use in clinical practice than other prognostic factors because of its high reproducibility.

In conclusion, our study showed that TICA was a significant risk factor for early‐onset CIP in patients with NSCLC and had a high reproducibility. We recommend that clinicians carefully watch patients with TICA, especially within three months of anti‐PD‐1 antibody administration, because of its high CIP risk. However, further studies are needed to elucidate whether the results of this study can be applied to patients receiving combination therapy.

## Disclosure

Dr Moda reports personal fees from Astra Zeneca and personal fees from Nippon Boehringer Ingelheim outside the submitted work.

Dr Saito reports grants from Chugai Pharmaceutical, grants from AtraZeneca, personal fees from Ono Pharmaceutical, personal fees from Nippon Boehringer Ingelheim, grants from MSD, and personal fees from Novartis Pharma, outside the submitted work.

Dr Usui reports personal fees from lecture fee outside the submitted work.

Dr Nakahara reports grants from Takeda, grants and personal fees from Eli Lilly, grants and personal fees from Bristol ‐Myers Squibb, personal fees from Ono Pharmaceutical Co., Ltd., personal fees from Boehringer Ingelheim, personal fees from Astrazeneca, and personal fees from MSD K.K., outside the submitted work.

Dr Kato reports grants and personal fees from Abbvie, grants and personal fees from Amgen, grants and personal fees from AstraZeneca, grants and personal fees from Boehringer Ingelheim, grants and personal fees from Bristol Myers Squibb, grants and personal fees from Chugai, grants and personal fees from Eli Lilly, grants and personal fees from Merck Biopharma, grants and personal fees from MSD, grants and personal fees from Novartis, grants and personal fees from Ono, grants and personal fees from Pfizer, grants and personal fees from Taiho, personal fees from Daiichi‐Sankyo, personal fees from F. Hoffmann‐La Roche, personal fees from Nippon Kayaku, personal fees from Nitto Denko, personal fees from Shionogi, personal fees from Sumitomo Dainippon, personal fees from Takeda, grants from Astellas, grants from Kyorin, grants from Kyowa‐Kirin, and grants from Regeneron, outside the submitted work.

The rest of the authors have nothing to disclose.
